# Smoking cessation and weight loss before ventral hernia repair – can we really justify this? A single center cohort study

**DOI:** 10.1007/s10029-026-03622-w

**Published:** 2026-03-21

**Authors:** Line Marker, Anna Fisker, Frederik Helgstrand

**Affiliations:** Department of Surgery, Zealand University Hospital, Køge, Denmark

**Keywords:** Ventral hernia repair, Preoperative optimization, Prehabilitation, Smoking cessation, Weight loss, Cohort study

## Abstract

**Purpose:**

Smoking and adiposity are risk factors for poor postoperative outcomes after hernia surgery. This study evaluated a real-world hospital-based prehabilitation program covering smoking cessation and weight loss prior to ventral hernia repair.

**Methods:**

In this retrospective single-center cohort study, patients enrolled in a non-standardized smoking cessation or weight loss program prior to ventral hernia repair between June 2021 and December 2024 were included. Patients in the smoking cessation program were offered counseling and motivational interviews by nurse specialists. Patients in the weight loss program received the same in addition to a target weight and dietary guidance. Follow-up occurred every 2–8 weeks according to patient preference. Success was defined as cessation of smoking for ≥ 6 weeks or reaching target weight followed by ventral hernia repair.

**Results:**

A total of 107 patients were identified: 12 (11.2%) participated in the smoking cessation program, 77 (72.0%) in the weight loss program, and 18 (16.8%) in both programs. Of these, 28 patients (26.2%) completed prehabilitation and underwent surgery, whereas 28 (26.2%) did not and remained in the program at the end of follow-up. A total of 27 (25.2%) patients dropped out, 14 (13.1%) were discontinued due to lack of progress, 8 (7.4%) were lost to follow-up, and 3 (2.8%) required emergency surgery. The median time from intervention to surgery was 325 days [IQR: 225.3, 496.5].

**Conclusion:**

Only one in four patients completed prehabilitation and underwent surgery. The program was resource-intensive, with substantial dropouts and failure rates. These results highlight the challenges associated with preoperative lifestyle modification prior to hernia surgery.

## Introduction

Ventral hernia repair (VHR) is one of the most common surgical procedures worldwide [1]. Despite its prevalence, ventral hernias remain challenging to manage and repair. VHR is associated with a substantial risk of postoperative complications, including surgical site infections (SSI), hernia recurrence, and reoperation [2]. The risk of complications varies depending on the type and size of the hernia, and whether it is a high-risk or low-risk repair [3]. The impact of patient comorbidities on postoperative outcomes has been widely discussed, and increasing attention has been directed toward optimization and patient prehabilitation prior to elective VHR [4].

Smoking and obesity are well-established risk factors for adverse postoperative outcomes after VHR, with up to a six-fold higher risk of surgical site occurrences (SSO) following open repair [5–7]. Smoking is further associated with an increased risk of hernia recurrence [8], and postoperative pulmonary complications after open surgery, contributing to higher 30-day mortality rates [9]. Evidence suggests that smoking cessation 4–8 weeks prior to surgery reduces postoperative morbidity and lowers the risk of wound complications [10, 11].

Obesity is a predictor for development of ventral hernias [1, 12], and an independent risk factor for postoperative complications, including SSI, readmission, nosocomial infections, abscess formation and recurrence following VHR [13–16]. The presence of these modifiable risk factors, combined with evidence that preoperative smoking cessation and weight loss improve outcomes in other surgical populations, has underscored the need for effective and standardized prehabilitation strategies for patients undergoing VHR [17, 18].

To reduce the risk of adverse postoperative outcomes, smoking cessation and weight loss are commonly considered mandatory before elective hernia surgery [19]. However, smoking cessation often requires structured support and guidance to be successful [20], and sustained weight loss is difficult to achieve [21, 22]. Consequently, prehabilitation may substantially prolong the time to surgery and potentially increase the risk of emergency repair in an already high-risk patient population. The aim of this study was to evaluate a smoking cessation and weight loss program based on patient instruction and motivational interviews prior to VHR, with a focus on feasibility within routine surgical outpatient care.

## Methods

This retrospective cohort study included patients aged ≥ 18 years who were enrolled in a smoking cessation and/or weight loss prehabilitation program prior to elective VHR. Patients were identified through the electronic health record (EHR) between June 2021 and December 2024. Smoking status and body mass index (BMI) were recorded at the initial outpatient visit.

Eligibility for prehabilitation was determined at a multidisciplinary team conference involving hernia surgeons and specialist nurses. Patients eligible for elective VHR who were current smokers and/or had obesity (BMI > 30 kg/m² or > 35 kg/m² depending on hernia complexity) were offered participation in the prehabilitation program.

The prehabilitation program was a pragmatic, guidance-based intervention embedded in routine surgical outpatient care and delivered using existing departmental resources, without additional personnel or structured lifestyle teams beyond nurse-led follow-up. Initial counselling by the treating surgeon focused on modification of surgical risk factors related to smoking and obesity and on defining the aims of prehabilitation. Individualized targets were set collaboratively based on baseline BMI, comorbidities, and patient preferences.

Follow-up was offered approximately 14 days after enrolment, with subsequent contacts scheduled flexibly, either by telephone or outpatient visits every 2–8 weeks, according to patient preference, progress, and need for support. Follow-up consisted of brief motivational interviews conducted by specialist nurses. The motivational interviews focused on goal clarification, review of progress, identification of barriers to adherence, and reinforcement of lifestyle targets. Follow-up was conducted either by telephone or during in-person outpatient visits.

Weight-loss prehabilitation included advisory dietary guidance using either a very low-calorie diet (5–6 meal-replacement powders/day) or a calorie-restricted diet (1400–1900 kcal/day). Adherence was patient driven. Referral to municipal smoking cessation or weight-loss programs were offered but voluntary and external to the hospital-based pathway. Written educational material on smoking cessation and dietary modification was distributed during outpatient visits.

No structured pharmacological weight loss protocol existed, but some patients independently initiated pharmacological weight-loss therapy (e.g., Semaglutide) outside the formal prehabilitation program. This was patient-driven and not offered or managed as part of the prehabilitation pathway.

Patients who achieved smoking cessation or reached their individualized weight target were offered surgery. Smoking cessation was verified using urine cotinine testing on the day of surgery. Successful prehabilitation was defined as achievement of smoking cessation for a minimum of six weeks and/or reaching the target weight followed by planned VHR.

Reasons for non-surgical management were extracted from the EHR and classified as dropouts, discontinuation due to lack of progress, lost to follow-up, or ongoing prehabilitation at the end of follow-up. Discontinuation due to lack of progress was determined at a multidisciplinary team conference involving hernia surgeons and specialist nurses.

Weight-related, health-related, and patient-related variables were collected from the EHR. The primary outcome was the proportion of patients who achieved smoking cessation and/or target weight and subsequently underwent planned VHR. Secondary outcomes included follow-up duration, number of patient contacts with the clinic, and occurrence of emergency surgery during the prehabilitation period.

### Statistics

Descriptive statistics were used to summarize patient characteristics, hernia characteristics, and outcomes. Continuous variables were presented as medians with interquartile ranges [Q1, Q3], and categorical variables as counts and percentages. Between-group comparisons of continuous variables were performed using the Mann-Whitney U test or Kruskal-Wallis test, as appropriate, with post hoc pairwise comparisons adjusted using the Benjamini-Hochberg method. Categorical variables were compared using Fisher’s exact test.

Exploratory univariable logistic regression analyses were performed to assess potential predictors of successful prehabilitation, including baseline BMI, age, sex, smoking, hernia type, and use of semaglutide. Results were reported as odds ratios with 95% confidence intervals. These analyses were considered exploratory due to the limited sample size.

Time to successful prehabilitation was analyzed using survival analysis. The event was defined as successful prehabilitation. Patients were censored at discontinuation of prehabilitation, emergency surgery, or end of follow-up (April 2025) if successful prehabilitation had not been achieved. Follow-up time was defined as time from initiation of prehabilitation to event or censoring. In addition, survival analysis was stratified by program type (smoking cessation, weight loss, or both) and compared using the log-rank test. A p-value < 0.05 was considered statistically significant.

## Results

A total of 107 patients were included in the study. Median age was 56 years [49, 62] and median follow-up time was 336 days [245, 513.5]. One of the patients who underwent surgery was initially considered for open abdominal wall reconstruction requiring a BMI < 30 kg/m². However, the surgical plan was revised to robot-assisted repair allowing a BMI < 35 kg/m², eliminating the need for preoperative weight loss. In accordance with the intention-to-treat principle, this patient was retained in the analyses. Patient demographics are summarized in Table 1.

**Table 1:** Demographics and outcomes of the patients enrolled in smoking cessation, weight loss, or combined programs

	All (*n* = 107)	Smoking cessation (*n *= 12)	Weight loss (*n *= 77)	Smoking cessation and weight loss (*n* = 18)
Age, years (Median [Q1, Q3])	56 [49, 62]	55 [48, 59]	56 [50, 63]	54 [47, 59]
Gender female, n (%)*	67 (62.6%)	8 (66.7%)	47 (61.0%)	12 (66.7%)
Hernia type, n (%)*				
Epigastric hernia	29 (27.1%)	1 (8.3%)	4 (5.2%)	0 (0.0%)
Umbilical hernia	42 (39.3%)	4 (33.3%)	31 (40.3%)	7 (38.9%)
Portside hernia	17 (15.9%)	0 (0.0%)	15 (19.5%)	2 (11.1%)
Incisional hernia	5 (4.7%)	7 (58.3%)	17 (22.1%)	5 (27.8%)
Parastomal hernia	14 (13.1%)	0 (0.0%)	10 (13.0%)	4 (22.2%)
EHS classification n (%)*				
M1, M2, M3	1 (0.9%)	-	-	1 (5.6%)
M2	16 (15.0%)	3 (25.0%)	12 (15.6%)	1 (5.6%)
M2, M3	33 (30.8%)	5 (41.7%)	21 (27.3%)	7 (38.9%)
M2, M3, M4	2 (1.9%)	1 (8.3%)	-	1 (5.6%)
M3	33 (30.8%)	1 (8.3%)	28 (36.4%)	4 (22.2%)
M3, M4	4 (3.7%)	2 (16.7%)	2 (2.6%)	-
L3	3 (2.8%)	-	3 (3.9%)	-
L4	1 (0.9%)	-	1 (1.3%)	-
Parastomal				
Type 1	7 (6.54%)	-	6 (7.8%)	1 (5.56%)
Type 2	5 (4.7%)	-	3 (3.9%)	2 (11.1%)
Type 4	2 (1.9%)	-	1 (1.3%)	1 (5.56%)
Loss of domain, n (%)*	2 (1.9%)	-	2 (2.6%)	-
Defect size (Median [Q1, Q3])				
Length	6.4 [3.3, 9.8]	6 [3.3, 8]	4 [2, 7]	6.4 [3.3, 9.8]
Width	4.8 [3, 6]	4.5 [2.8, 6]	4 [2.5, 6.9]	4.8 [3, 6]
Medical conditions, n (%)*				
Diabetes mellitus type II	21 (19.6%)	1 (8.3%)	18 (23.4%)	2 (11.1%)
Hypertension	51 (47.7%)	4 (33.3%)	37 (48.1%)	10 (55.6%)
COPD/asthma	36 (33.6%)	7 (58.3%)	23 (29.9%)	6 (33.3%)
Heart disease	19 (17.8%)	1 (8.3%)	13 (16.9%)	5 (27.8%)
Hyperlipidemia	29 (27.1%)	2 (16.7%)	21 (27.3%)	6 (33.3%)
ASA score n (%)*				
2	56 (51.4%)	9 (75%)	39 (50.5%)	8 (44.4%)
3	51 (47.7%)	3 (25%)	38 (49.4%)	10 (55.6%)
Initial body mass index, kg/m2 (Median [Q1, Q3])	-	-	40.3 [37.7, 43.8]	37.3 [35.7, 39.2]
Follow-up Body mass index, kg/m2 (Median [Q1, Q3])	-	-	37.9 [35.0, 41.8]	34.8 [33.3, 37.2]
Weight loss, kg (Median [Q1, Q3])	-	-	5.2 [1, 10.5]	4.5 [2.2, 9.5]
Weight change per. 100 days, kg (Median [Q1, Q3])	-	-	1.62 [0.28, 3.28]	1.35 [0.46, 2.27]
Numbers of contacts, n (Median [Q1, Q3])	6 [3, 8]	3 [1.5, 4]	7 [4, 9]	6 [4, 9]
Emergency repair, n (%)*	3 (2.8%)	0	3 (3.9%)	0

*% of total subgroup population

**Fig. 1:**
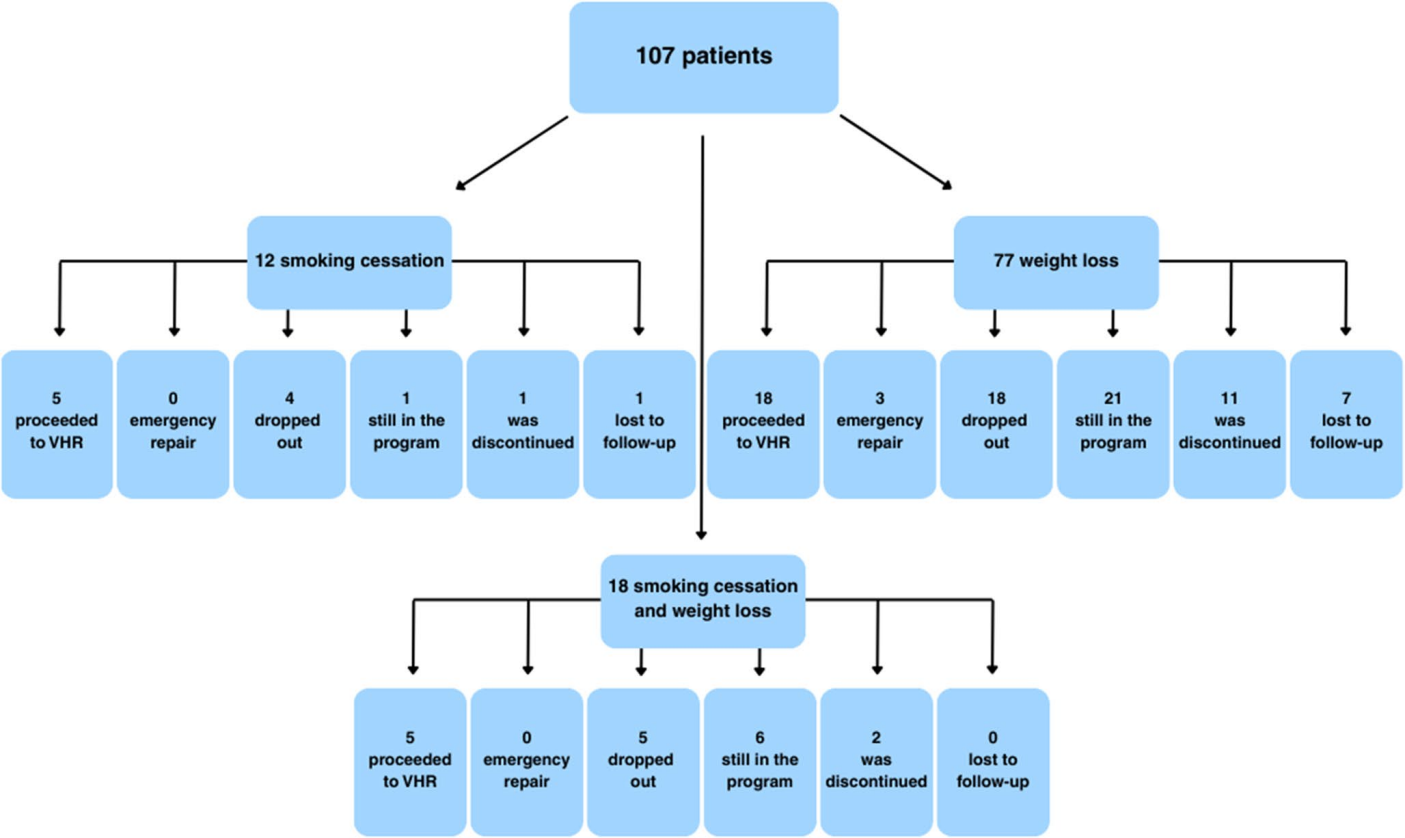
Patient outcomes and distribution by intervention group

Twelve patients participated in the smoking cessation program, 77 in the weight loss program, and 18 in both programs. There was no difference in age (*p* = 0.949) or sex (*p* = 0.453) between groups. Patient flow and outcome are illustrated in Fig. 1. 

Overall, 28 patients (26.2%) completed the prehabilitation and underwent planned VHR (Table 2). All patients who achieved successful prehabilitation within the follow-up period proceeded to surgery.

**Table 2:** Characteristics and outcomes of patients who proceeded to planned ventral hernia repair by intervention group

	All	Smoking cessation	Weight loss	Smoking cessation and weight loss
Proceeded to ventral hernia repair, n (%)*	28 (26.2%)	5 (41.7%)	18 (23.4%)	5 (27.8%)
Hernia type, n (%)**				
Epigastric hernia	1 (3.6%)	1 (20.0%)	0 (0.0%)	0 (0.0%)
Umbilical hernia	9 (32.1%)	2 (40.0%)	5 (27.9)	2 (40.0%)
Port side hernia	5 (17.9%)	0 (0.0%)	5 (27.8%)	0 (0.0%)
Incisional hernia	12 (42.9%)	2 (40.0%)	7 (38.9%)	3 (60.0%)
Parastomal hernia	1 (3.6%)	0 (0.0%)	1 (5.6%)	0 (0.0%)
EHS classification n (%)**				
M1, M2, M3	1 (3.6%)	-	-	1 (20.0%)
M2	7 (25.0%)	1 (20.0%)	6 (33.3%)	-
M2, M3	9 (32.1%)	1 (20.0%)	5 (27.9%)	3 (60.0%)
M2, M3, M4	1 (3.6%)	-	-	1 (20.0%)
M3	6 (21.4%)	1 (20.0%)	5 (27.9%)	-
M3, M4	3 (10.7%)	2 (40.0%)	1 (5.6%)	-
L3	-	-	-	-
L4	-	-	-	-
Parastomal				
Type 1	1 (3.6%)	-	1 (5.6%)	-
Type 2	-	-	-	-
Type 4	-	-	-	-
Loss of domain n (%)**	0	0	0	0
Defect size (Median [Q1, Q3])				
Length	6.5 [3, 10.5]	3.4 [3, 8]	4.6 [2, 7.8]	12 [12, 12]
Width	4.5 [3, 7.3]	4 [3, 5]	4.5 [3, 7.8]	6 [3, 7]
Follow-up, days (Median [Q1, Q3])	325 [225.3, 496.5]	279 [230, 371]	325 [216, 490]	340 [244, 604]
Initial body mass index, kg/m2 (Median [Q1, Q3])	-	-	37.9 [36.1, 39.0]	36.0 [35.8, 36.0]
Follow-up Body mass index, kg/m2 (Median [Q1, Q3])	-	-	34.3 [32.6, 35.5]	34.5 [33.4, 35.0]
Weight loss, kg (Median [Q1, Q3])	-	-	9.8 [8.0, 12.0]	4[3.7, 5.0]
Numbers of healthcare contacts, n (Median [Q1, Q3])	5 [3, 7.3]	3 [1, 4]	5 [3, 9.3]	5 [5, 8]
Postoperative outcomes, n (%)**				
SSI	2 (7.1%)	0 (0%)	1 (5.6 %)	1 (20.0%)
SSO	2 (7.1%)	0 (0%)	4 (22.2%)	1 (20.0%)
Readmission	3 (10.7%)	0 (0%)	2 (11.1%)	0 (0.0%)
Recurrence	1 (3.6%)	0 (0%)	1 (5.6%)	0 (0.0%)
Length of stay (Median [Q1, Q3])	0.5 [0, 5]	0 [0, 0]	1 [0, 5]	1 [0, 6]
Type of procedure, n (%)**				
Open	11 (39.3%)	1 (20.0%)	8 (44.4%)	2 (40.0%)
Laparoscopic	3 (10.7%)	1 (20.0%)	2 (11.1%)	0 (0.0%)
Robot assisted	14 (50.0%)	3 (60.0%)	8 (44.4 %)	3 (60.0%)
Converted to open surgery	1 (3.6%)	0 (0.0%)	1 (5.6 %)	0 (0.0%)

*% of total subgroup population

**% of subgroup population proceeded to ventral hernia repair

**Fig. 2:**
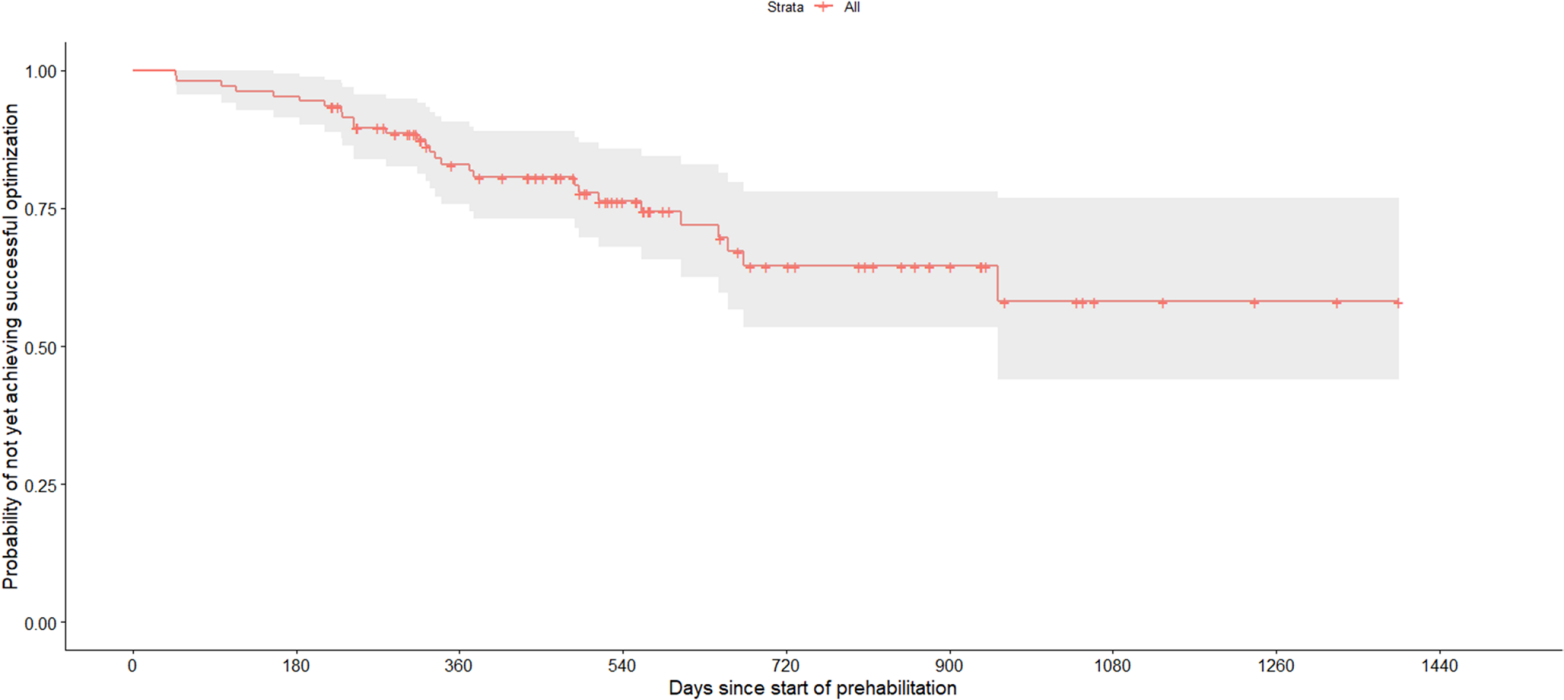
Kaplan-Meier curve showing time to successful prehabilitation, defined as achievement of smoking cessation and/or target weight followed by elective ventral hernia repair

**Fig. 3:**
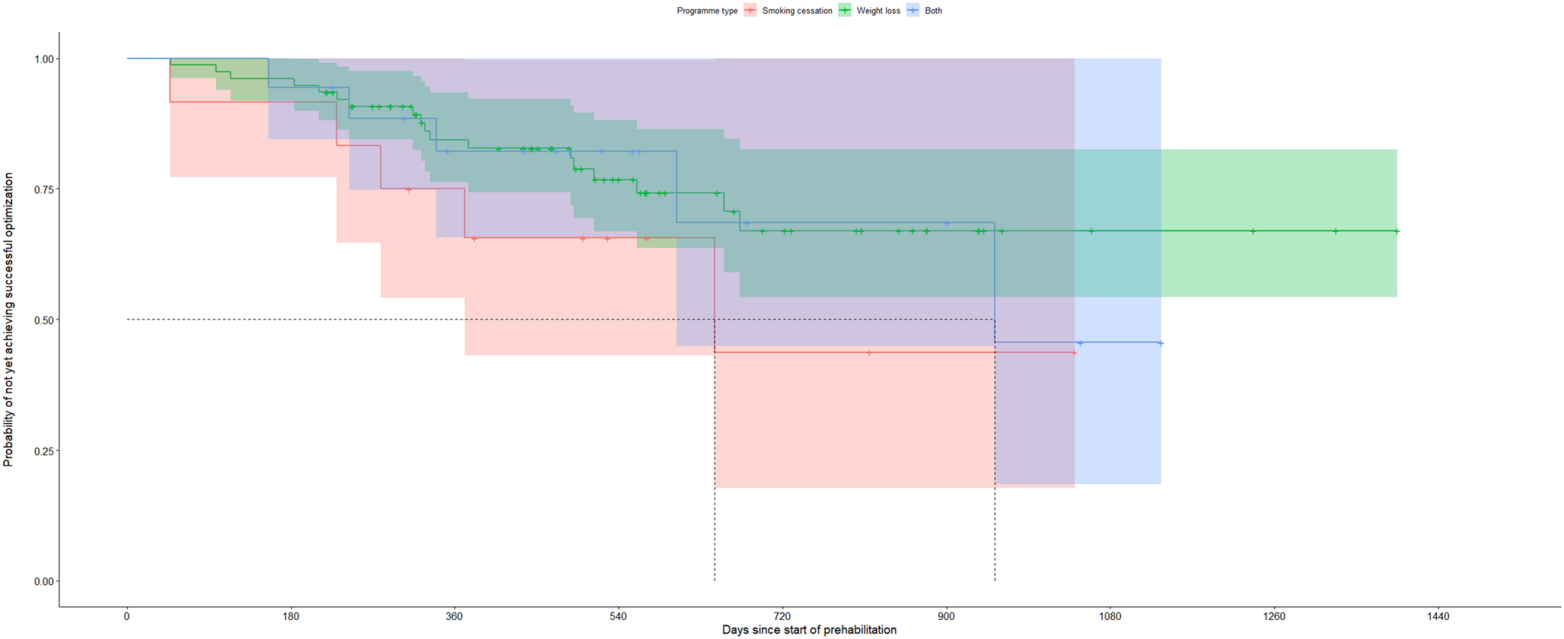
Kaplan-Meier curves stratified by program type (smoking cessation, weight loss, or both) showing time to successful prehabilitation

Median time from the start of prehabilitation to surgery was 325 days [225.3, 496.5]. The Kaplan-Meier curve demonstrated a gradual achievement of successful prehabilitation over time with most successes occurring within the first year (Fig. 2). In the stratified survival analysis, no statistically significant differences were observed between program types (smoking cessation, weight loss, or both, *p* = 0.40) (Fig. 3). Subgroup sizes were small, particularly for patients enrolled in smoking cessation alone and in both programs. Median number of healthcare contacts among patients who completed prehabilitation was 5 [3, 7.3].

Among patients undergoing elective VHR, SSI and SSO each occurred in 7.1%, and hernia recurrence in 3.6%. Median length of stay was 0.5 days (0, 5).

At the end of follow-up, 77 patients did not complete the prehabilitation program: 7 (58.3%) from the smoking cessation group, 57 (74.0%) from the weight loss group, and 13 (72.2%) from the combined group (Table 3). For these patients, the median follow-up time was 343 days [271.5, 514], and the median number of contacts was 7 [4, 8]. There was no significant difference in either age or sex between patients who proceeded to surgery and those who did not (Tables 4 and 5).

**Table 3:** Characteristics and outcomes of patients who did not complete the prehabilitation program by intervention group

	All	Smoking cessation	Weight loss	Smoking cessation and weight loss
Total, n (%)*	77 (73.8%)	7 (58.3%)	57 (74.0%)	13 (72.2%)
Still in the program, n (%)*	28 (26.2%)	1 (8.3%)	21 (27.3%)	6 (33.3%)
Dropped out, n (%)*	27 (25.2%)	4 (33.3 %)	18 (23.4%)	5 (27.8%)
Discontinued due to lack of progress, n (%)*	14 (13.1%)	1 (8.3%)	11 (14.3%)	2 (11.1%)
Lost to follow-up, n (%)*	8 (7.4%)	1 (8.3%)	7 (9.1%)	0
Hernia type, n (%)**				
Epigastric hernia	4 (5.2%)	0 (0.0%)	4 (7.0%)	0 (0.0%)
Umbilical hernia	31 (40.3%)	2 (28.6%)	24 (42.1%)	5 (38.5%)
Port side hernia	12 (15.6%)	0 (0.0%)	10 (17.5%)	2 (15.4%)
Incisional hernia	17 (22.1%)	5 (71.4%)	11 (19.3%)	2 (15.4%)
Parastomal hernia	13 (16.9%)	0 (0.0)	8 (14.0%)	4 (30.8%)
EHS classification n (%)**				
M1, M2, M3	-	-	-	-
M2	9 (11.7%)	2 (28.6)	6 (10.5%)	1 (7.7%)
M2, M3	22 (28.6%)	4 (57.1%)	14 (24.6)	4 (30.8%)
M2, M3, M4	1 (1.3%)	1 (14.3%)	-	-
M3	27 (35.1%)	-	23 (40.4)	4 (30.8%)
M3, M4	1 (1.3%)	-	1 (1.8%)	-
L3	3 (3.9%)	-	3 (5.3%)	-
L4	1 (1.3%)	-	1 (1.8%)	-
Parastomal				
Type 1	6 (7.8%)	-	5 (8.8%)	1 (7.7%)
Type 2	5 (6.5%)	-	3 (5.3%)	2 (15.4%)
Type 4	2 (2.6%)	-	1 (1.8%)	1 (7.7%)
Loss of domain, n (%)*	2 (2.6%)	2 (28.6%)	0	0
Defect size (Median [Q1, Q3])				
Length	4 [2, 7]	6 [5.3, 9.8]	3.5 [2, 6]	4.5 [2, 7.2]
Width	4 [2, 6]	5.5 [3, 8]	3.3 [2, 5]	4.5 [3, 5.5]
Follow-up, days (Median [Q1, Q3])	343 [270, 502]	321 [257, 408]	336 [264.5, 514]	436 [316, 521]
Initial Body mass index, kg/m2 (Median [Q1, Q3])	-	-	40.5 [38.5, 44]	38.4 [35.7, 40.7]
Follow-up Body mass index, kg/m2 (Median [Q1, Q3])	-	-	39.7 [36.3, 43.4]	34.9 [33.2, 38.4]
Weight loss, kg (Median [Q1, Q3])	-	-	3 [-1, 7.6]	7 [2, 11.2]
Numbers of healthcare contacts, n (Median [Q1, Q3])	7 [4, 8]	3 [2, 3.5]	7 [4, 8]	7 [4, 9]

*% of total subgroup population

**% of subgroup population not completing prehabilitation

**Table 4:** Sex distribution of patients by intervention group and surgical outcome

	Women surgery (n)	Women no surgery (n)	Men surgery (n)	Men no surgery (n)	*P-*value
Smoking	3	5	2	2	1.000
Weight	10	36	8	21	0.589
Both	2	10	3	3	0.268

**Table 5:** Age distribution of patients by intervention group and surgical outcome

	No surgery (n)	Age – (Median [Q1, Q3])	Surgery (n)	Age – (Median [Q1, Q3])	Adjusted *p*-value
Smoking	7	59.0 [55.6, 62.2]	5	50.2 [40.4, 54.4]	0.144
Weight	57	56.6 [50.7, 63.5]	18	59.8 [53.2, 63.8]	0.417
Both	13	55.0 [48.7, 59.7]	5	53.9 [47.6, 60.2]	1.000

In univariable logistic regression analyses, lower baseline BMI was significantly associated with successful prehabilitation (OR 0.86 per BMI unit, 95% CI 0.78–0.94, p = 0.002). Thirty patients (31.6%) in the weight-loss program reported semaglutide use during prehabilitation (Table 6). Of these, 8 patients (27.6%) achieved their target weight and underwent surgery, two required emergency surgery, and one remained in prehabilitation at the end of follow-up. Age, sex, smoking status, and use of semaglutide were not significantly associated with successful prehabilitation (Table 7).

**Table 6:** Semaglutide vs. non-semaglutide

	Semaglutid (n = 30)	Non-semaglutid (n = 65)	*p*-value
Age, years, (Median [Q1, Q3])	56 [51, 61]	56 [49, 63]	0.651
Gender female, n (%)	18 (60.0%)	41 (63.1%)	0.952
Initial Body mass index, kg/m2 (Median [Q1, Q3])	39.2 [37.3, 41.2]	39.6 [36.8, 43.0]	0.776
Numbers of healthcare contacts, n (Median [Q1, Q3])	7.5 [5, 10]	6 [4, 8]	0.098
Reached weight goal and underwent elective VHR, n (%)	8 (26.7%)	15 (23.1%)	0.798
Emergency repair, n (%)	2 (6.7%)	1 (1.5%)	0.234

**Table 7:** Univariable logistic regression analyses of predictors for successful prehabilitation

	OR	95% CI	*p*-value
Age (per year)	1.00	0.96 – 1.04	0.939
Gender female	1.67	0.69 – 4.03	0.252
Initial BMI (per unit)	0.86	0.78 – 0.94	0.002
Active smoker	1.48	0.60 – 3.65	0.389
Semaglutide	1.04	0.38 – 2.64	0.941
Hernia type			
Epigastric hernia	0.92	0.04 – 7.23	0.941
Incisional hernia	2.59	0.92 – 7.55	0.074
Parastomal hernia	0.28	0.01 – 1.73	0.252
Port side hernia	1.53	0.40 – 5.41	0.516

Three patients (2.8%) required emergency VHR during the prehabilitation period. One patient presented with small bowel obstruction, one with strangulated preperitoneal adipose tissue, and one with an incarcerated hernia containing small bowel loops but without clinical or radiological evidence of obstruction. Baseline BMI for these patients was 48.8, 41, and 45.3, respectively. Two patients had an umbilical hernia, and one had an epigastric hernia. Time from initiation of prehabilitation to emergency surgery was 594, 708, and 867 days. The patient with an epigastric hernia continued in the weight loss program postoperatively for treatment of an umbilical hernia.

## Discussion

Preoperative prehabilitation to achieve smoking cessation and reduce excessive weight prior to VHR is recommended to improve outcomes after VHR [4]. In this study, we assessed the consequences of a smoking cessation and weight loss program based on patient instruction and motivational interviews. Only one in four patients achieved their prehabilitation goals and underwent surgery (26.2%). Among these patients, the median follow-up time was 325 days, with a median of five contacts with the clinic. Although the program was deliberately designed as a simple and pragmatic intervention, it proved both time-consuming and resource-intensive due to limited patient progress. There was no statistically significant difference in follow-up duration between patients who proceeded to VHR and those who did not complete the program, suggesting that time spent in prehabilitation was not associated with the likelihood of undergoing surgery. A lower baseline BMI was significantly associated with successful prehabilitation among the patients enrolled in the weight loss program, indicating that patients with less severe obesity may be more likely to achieve preoperative weight-loss targets within a pragmatic prehabilitation framework.

Our findings align with the existing literature highlighting the challenges of prehabilitation. A case control study reported that 19% of advised patients stopped smoking prior to VHR compared with 2% in a non-advised cohort (*P* < 0.01). However, fewer than one in five patients in the advised group succeeded, indicating that even targeted interventions may have limited impact [20]. Similarly, a randomized controlled trial including patients undergoing primary hernia repair, laparoscopic cholecystectomy, or hip or knee replacement demonstrated higher smoking cessation rates following individual counseling and nicotine substitution compared with standard counseling (40% vs. 2%; *P* < 0.001) [23]. Nicotine substitution may facilitate smoking cessation, and when smoking cessation is combined with transdermal nicotine administration, cutaneous microvascular perfusion normalizes within seven days [24]. Moreover, smokers who use nicotine patches for four weeks or longer have a reduced incidence of wound infections compared with continued smokers [25]. Nonetheless, verification of smoking abstinence remains challenging, as standard urine cotinine testing cannot distinguish between continued smoking and nicotine substitution therapy.

Previous weight loss initiatives have shown mixed results. In an evaluation of a free Weight Management Navigator (WMN) program for obese patients seeking VHR, enrolment was low and only 10% of participants achieved sufficient weight loss for surgery [26]. In contrast, a very low-calorie diet-based program achieved substantial short-term weight loss, with 50% of patients proceeding to surgery [27]. Another study compared prehabilitation to standard counseling in VHR candidates with BMI 30–40 kg/m². Prehabilitation included nutrition, physiotherapy, and a hernia navigator, and led to more patients reaching weight loss goals (22.2% vs. 10.3%), though this was not statistically significant [28].

In our study, only one in four participants achieved their goal, despite the assumption that they would be highly motivated by the prospect of eventually undergoing surgery. Patient expectations may represent a critical barrier to successful prehabilitation. Previous work has shown that a substantial proportion of ventral hernia patients expect to undergo surgery, and that patients enrolled in prehabilitation may experience decisional regret even when optimization is agreed upon to improve outcomes [29]. Misaligned expectations and limited awareness of the need for risk factor modification may therefore reduce adherence. In contrast, weight loss was successfully achieved in 24 of 25 patients (mean BMI 49 ± 10 kg/m²) prior to complex VHR through the use of a protein-sparing modified fast combined with structured goal setting, where patients first defined their own goals before progressing to surgeon-defined targets [30]. Patient education and involving patients in goal setting may improve adherence [31], although motivation is also influenced by broader healthcare system factors. Universal access to healthcare may facilitate engagement, whereas insurance-based systems may delay or restrict treatment, as illustrated by the Oregon Health Insurance Experiment [32].

Achieving sustained weight loss and smoking cessation remains challenging. Weight loss is biologically and behaviorally difficult to maintain due to adaptive metabolic and hormonal responses as well as environmental factors [33]. Only about 20% of patients succeed in maintaining ≥ 10% weight loss for at least one year [33, 34]. Likewise, smoking cessation is difficult due to withdrawal mechanisms with unassisted quit attempts yielding long-term success in under 10% [35]. These challenges underscore the complexity of achieving durable risk factor modification, even among motivated patients, and the long-term implications of postoperative weight gain or resumed smoking on hernia recurrence remain uncertain.

Smoking and obesity are more prevalent among individuals with low socioeconomic status, including those with lower levels of education and income [36, 37]. Limited resources and social challenges may decrease the likelihood of successful smoking cessation or weight loss, even when interventions are provided free of charge and supported by healthcare professionals. This presents a significant challenge for prehabilitation strategies that require substantial lifestyle changes to optimize surgical outcomes [36, 37]. Although pharmacological weight loss therapies have gained popularity [38], access remains limited due to high costs, disproportionately affecting patients with lower socioeconomic status [39]. In our cohort, one-third of patients who achieved weight loss goals reported use of pharmacological agents. However, no difference in outcomes was observed between users and non-users. Importantly, pharmacological therapy was patient-initiated and not part of the prehabilitation program, reflecting a deliberate focus on a hospital-based, cost-effective intervention that does not rely on treatments with limited accessibility.

Much of the evidence linking smoking and obesity to adverse postoperative outcomes is derived from open surgical procedures [13, 14, 40]. In this study, postoperative complications were infrequent, with low rates of SSI, SSO, readmission, and hernia recurrence. While prehabilitation aims to improve postoperative outcomes, these complications are presented descriptively to enhance transparency rather than to imply benefit or harm associated with the prehabilitation pathway.

To place the present findings in a broader clinical context, advances in minimally invasive VHR have been proposed as a potential way to mitigate some of the risks traditionally associated with smoking and obesity [41]. While laparoscopic repair of ventral or incisional hernias with intraperitoneal mesh (laparoscopic IPOM) has long been considered the gold standard [42], concerns regarding mesh-related complications have led to increased adoption of minimally invasive preperitoneal and robotic techniques [43–46]. Robotic techniques make it possible to treat even complex hernias with a minimally invasive approach and show advantages with lower readmission rates, shorter length of stay, less SSI, lower postoperative pain, better functional recovery and improved cosmesis, compared with conventional laparoscopic IPOM and open repairs [47–49]. Retrospective studies suggest that smoking and obesity may be less strongly associated with short-term wound complications following laparoscopic or robotic preperitoneal mesh repair compared with open surgery [41, 50]. Similarly, studies examining patients with elevated BMI undergoing minimally invasive repair have reported comparable rates of recurrence, seroma, hematoma, and SSI to those observed in patients with lower BMI [51]. These findings were not explored in our study, but suggest that while prehabilitation remains important, failure to achieve prehabilitation goals should not necessarily preclude elective VHR in all patients.

A concern when delaying VHR is the risk of small bowel obstruction and the potential need for emergency surgery. In the present study, three patients (2.8%) required emergency repair, due to strangulated hernias, and one of these patients presented with a small bowl obstruction. All three patients had severe obesity and prolonged prehabilitation periods exceeding the median follow-up time of 336 days. This suggests that a long follow-up period is necessary to obtain a representative overview of the risk of emergency surgery. Although these cases represent outliers, they illustrate potential risks associated with extended delays and underscore the need for individualized decision-making [26, 52]. Patients with severe symptoms or at high risk of incarceration may benefit from timely elective minimally invasive repair regardless of success with prehabilitation goals, in order to avoid emergency surgery and symptom progression [53].

This study has limitations. The retrospective and descriptive design restrict the ability to establish causal relationships. Reliance on EHR data may have resulted in incomplete case identification. The prehabilitation program varied between patients. This heterogeneity may limit interpretability, as completion rates may reflect program design rather than feasibility alone. However, the prehabilitation program was deliberately designed as a low-resource, pragmatic intervention integrated into routine surgical care, reflecting what may be feasible and scalable in high-volume surgical settings. Our findings also reflect real-world hospital-based practice as this program has been implemented within the study center since 2021. Furthermore, it was not possible to gather detailed information about why patients dropped out. Variation in medical staff at the outpatient clinic, ranging from highly specialized hernia surgeons to junior doctors in their first year of basic clinical training could influence patient communication. This could be interpreted as a limitation or a strength as it reflects daily practice. Future prospective studies are needed to define optimal prehabilitation strategies for high-risk patients. Given the difficulty of achieving sustained smoking cessation and weight loss, surgical strategies that compensate for these risk factors may represent a pragmatic alternative in selected patients [54].

## Conclusion

This retrospective cohort study demonstrates limited success and adherence to smoking cessation and weight loss interventions prior to ventral hernia repair in a real-world clinical setting. The findings highlight the substantial challenges associated with preoperative lifestyle modification and suggest that current prehabilitation goals may be difficult to achieve for a large proportion of patients.
